# Effect of the Polyketone Aromatic Pendent Groups on the Electrical Conductivity of the Derived MWCNTs-Based Nanocomposites

**DOI:** 10.3390/polym10060618

**Published:** 2018-06-05

**Authors:** Nicola Migliore, Lorenzo Massimo Polgar, Rodrigo Araya-Hermosilla, Francesco Picchioni, Patrizio Raffa, Andrea Pucci

**Affiliations:** 1 Department of Chemical Engineering, University of Groningen, Nijenborgh 4 9747 AG, The Netherlands; n.migliore@student.rug.nl (N.M.); lmpolgar@hotmail.com (L.M.P.); f.picchioni@rug.nl (F.P.); p.raffa@rug.nl (P.R.); 2 Department of Chemistry and Industrial Chemistry, University of Pisa, Via Giuseppe Moruzzi, 13, 56124 Pisa (PI), Italy; 3 Dutch Polymer Institute (DPI), P.O. Box 902, 5600 AX Eindhoven, The Netherlands; 4 Programa Institucional de Fomento a la Investigación, Desarrollo e Innovación, Universidad Tecnológica Metropolitana, Ignacio Valdivieso 2409, P.O. Box 8940577, San Joaquín, 8940000 Santiago, Chile; rodrigo.araya@utem.cl

**Keywords:** functionalized polyketones, MWCNTs nanocomposites, electrically conductive plastics

## Abstract

Electrically conductive plastics with a stable electric response within a wide temperature range are promising substitutes of conventional inorganic conductive materials. This study examines the preparation of thermoplastic polyketones (PK30) functionalized by the Paal–Knorr process with phenyl (PEA), thiophene (TMA), and pyrene (PMA) pendent groups with the aim of optimizing the non-covalent functionalization of multiwalled carbon nanotubes (MWCNTs) through π–π interactions. Among all the aromatic functionalities grafted to the PK30 backbone, the extended aromatic nuclei of PMA were found to be particularly effective in preparing well exfoliated and undamaged MWCNTs dispersions with a well-defined conductive percolative network above the 2 wt % of loading and in freshly prepared nanocomposites as well. The efficient and superior π–π interactions between PK30PMA and MWCNTs consistently supported the formation of nanocomposites with a highly stable electrical response after thermal solicitations such as temperature annealing at the softening point, IR radiation exposure, as well as several heating/cooling cycles from room temperature to 75 °C.

## 1. Introduction

Aliphatic polyketones (PKs) are terpolymers typically produced from carbon monoxide and unsaturated hydrocarbon monomers. They have not found widespread application in industry and everyday life yet, but their easy functionalization confer these materials with great potentiality [1,2,3]. For example, the scalable and mild Paal–Knorr functionalization reaction with N-functionalized amines yields pyrrolic moieties with a pendent functional group as in a graft polymer, and, in turn, tunable thermo-mechanical properties to desired values that are close to those of commodity thermoplastic polymers [4]. Particularly noteworthy is the use of furan-grafted PK, which promotes the preparation of a thermo-reversible crosslinked polymer network by means of Diels–Alder (DA) and retro-DA sequences employing conventional heating procedures [5,6,7,8,9,10]. These features were found remarkable for endowing functionalized PK with intrinsic self-healing characteristics and the ability of being mended and recycled (according to a “cradle to cradle” approach) in order to prolong their use after their first service life [3,11]. As far as smart materials are concerned, we have recently demonstrated the potentiality offered by functionalized PK in achieving flexible nanocomposites containing well-distributed, exfoliated, and undamaged multi-walled carbon nanotubes (MWCNTs) [12]. MWCNTs are graphitic monodimensional materials with multiple exceptional properties that have supported the virtues of their incorporation into polymeric matrices to produce high-strength, lightweight, and high-performance nanocomposites for a multitude of applications [13,14,15,16,17]. Thermoplastic polymers such as PK were found to be attractive supporting materials for MWCNT since they can be easily processed and fabricated into several solid-state forms required for different applications [18,19,20]. The percolative network was found to be poised enough even at high temperatures thanks to the effective stabilization of MWCNTs provided by the PK polymer matrix, but a resistivity–temperature profile with a negative temperature coefficient was found [12]. Although this property suggests the utilization of the nanocomposites as temperature sensors thanks to the semiconducting properties of MWCNTs, plastic polymers with electrically conductive features often require a stable electric response within a relatively wide range of temperatures [21,22,23]. Also, stability towards other external solicitations such as mechanical and chemical stresses is often required [24,25,26,27,28,29,30,31]. This is generally achieved by using a high content of the graphitic filler (i.e., well above the percolation threshold) or extensive annealing procedures in order to render the percolative pathways within the polymer matrix fixed and unalterable by external solicitations such as thermal stress [32]. However, shortcomings in these approaches suggest that the optimization and the maximization of the polymer/MWCNTs interactions at the interface is required, for example, by means of effective secondary interactions provided by non-covalent functionalization approaches. These approaches have proven to be highly effective and promising for the exfoliation and dispersion of MWCNTs in several polymer matrices as a consequence of being easily scalable, reversible, and preserving the structural integrity of the graphitic network [33]. The latter is a fundamental requisite to preserve the electronic properties of CNTs [34,35,36,37]. The most promising candidates to perform this task are the extended polycyclic aromatic groups as they, because of their structure, effectively interact with the graphitic surface of MWCNTs. Once incorporated within the polymer matrix by grafting procedures, they definitely exert their features, thus potentially providing a plastic nanocomposite with extremely stable percolation pathways and an electric response that is unaffected by thermal stress [38,39].

**Scheme 1:**
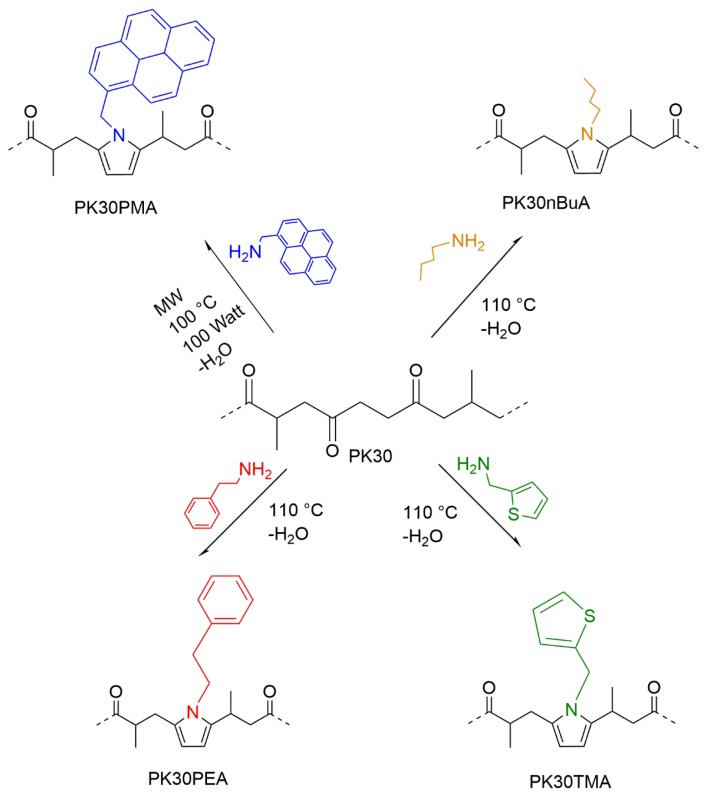
Schematic representation of modified polyketones (PKs) with different amino-compounds (nBuA: *n*-butylamine, TMA: 2-thiophenemethylamine, PEA: 1-phenylethylamine, PMA: 1-pyrenemethylamine).

In keeping with this rationale, the present work focuses on the preparation of various functionalized PK bearing aromatic pendent groups such as pyrene, thiophene, and benzene, plus a non-aromatic alkyl group as a reference, by means of the Paal–Knorr process (Scheme 1). The polymers were used as a supporting matrix to form nanocomposites containing different amounts of MWCNTs. The structural features of the nanocomposites as well as their electrical conductivity were analyzed and discussed in terms of the nature of the pendent aromatic moiety, filler content, and electric response towards thermal stress.

## 2. Materials and Methods

The alternating aliphatic polyketone (PK30; CO, ethylene, propylene: 50, 30, and 20 mol %, respectively, *M*_W_ = 2687 g/mol) was synthesized according to a reported procedure [40]. 2-thiophenemethylamine (TMA, pure), *n*-butylamine (nBuA, ≥99%), phenylethylamine (PEA, 99%), and 1-pyrenemethylamine hydrochloride (PMA, 95%) were purchased from Sigma-Aldrich (Darmstadt, Germany) and freshly distilled before use. Multi-walled carbon nanotubes (MWCNTs, Sigma-Aldrich, as-produced cathode deposit, >7.5% MWCNT basis, O.D. × L 7–15 nm × 0.5–10 μm) were used as an electrically conductive filler and used as received. Dimethylsulfoxide (DMSO, Acros, 99.7%), ammonia (Sigma-Aldrich, ca. 25), toluene (anhydrous, 99.8%, Sigma-Aldrich,), tetrahydrofuran (THF ≥ 99.9, Sigma-Aldrich), and methanol (Sigma-Aldrich, anhydrous, 99.8%) were used as received. Dimethyl sulfoxide-d6 (DMSO-d6, anhydrous, 99.9 atom % D, Sigma-Aldrich) was used as a deuterated solvent for ^1^H-NMR studies.

### 2.1. Functionalization of Alternating PK30 with Different Amino-Compounds

Typically, 20 g of PK30 was added in a sealed 250 mL round-bottomed glass reactor with a reflux condenser, a U-type anchor impeller, and an oil bath for heating. An equimolar amount of the desired amine (*n*-butylamine, phenylethylamine, 2-thiophenemethylamine) was added dropwise to the reactor in the first 20 min. The reaction was performed at 110 °C under vigorous stirring for 4.5 h. After the reaction, the mixture was dissolved in chloroform (around 230 mL) and washed several times with deionized Milli-Q water to remove unreacted amine, and then it was dried in a vacuum oven at 70 °C for 24 h. The conversion of amine modification was determined by elemental analysis. The reaction between PK30 and 1-pyrenemethylamine (in molar ratio between the 1,4-dicarbonyl groups of the PK30 and the amino groups, aiming at a 40% carbonyl conversion) was carried out using toluene as a solvent in a 100 mL round-bottomed flask equipped with a reflux condenser and using a microwave apparatus at 100 °C, 100 watts, with vigorous stirring for 3 h. After the reaction, the mixture was dried in a vacuum oven for 24 h. The samples in Table 1 were coded indicating the type of amine (n-BuA for butylamine, TMA for 2-thiophenemethylamine, PEA for phenylethylamine and PMA for 1-pyrenemethylamine) and the desired carbonyl conversion (X_CO_). Carbonyl conversion (X_CO_ in %) of PK was calculated using Equation (1), based on the calculation reported in literature [6]:

(1) $$X_{\mathrm{CO}}=\frac{2\;\frac{\%N}{M_{N}}}{\frac{\frac{\%C}{M_{C}}-\frac{\%N}{M_{N}}\cdot n_{y}^{c}}{n_{x}^{c}}+2\frac{\%N}{M_{N}}}\cdot 100$$

%N, %C: weight of nitrogen and carbon on 100 g of the final product, respectively;
M_N_: M_C_ atomic weights of nitrogen and carbon;
$n_{x}^{c}$: average number of carbons in the unmodified carbonyl repeating unit;
$n_{y}^{c}$: average number of carbons in the pyrrolic repeating unit.

The amine conversion (X_amine_ in %) was calculated from the moles of amine (butylamine, 2-thiophenemethylamine, phenylethylamine, and 1-pyrenemethylamine) in the feed (mol_amine_) using Equation (2):

(2) $$\chi_{\mathrm{amine}}=\frac{\frac{\%N}{M_{N}}}{{\mathrm{mol}}_{\mathrm{amine}}}\times 100$$

%N: weight of nitrogen on final product;
M_N_: atomic weights of nitrogen, 14.01 g/mol;
mol_amine_: moles of amine in the feed.

### 2.2. Mixing of TMA/PEA/PMA Modified PK30 with Conductive Fillers

In a common recipe, approximately 2.1 g of modified PK30 (PK30TMA, PK30PEA, or PK30PMA) was dissolved in approximately 35 mL of toluene. The required wt % of MWCNTs (Table 1) was mixed with the same solvent and sonicated in a bath for 30 min and then poured to the polymer solution in a round-bottomed flask at 50 °C for 24 h under stirring. Then, the mixture was rotary evaporated and finally transferred into a vacuum oven (70 °C for 48 h) to ensure the complete removal of the solvent. Rectangular solid samples with different dimensions were prepared by compression-molding at 150 °C for 30 min at 40 bar to ensure full homogeneity. Electrical measurements were carried out on fresh samples or on annealed samples at 75 °C for 10 min.

### 2.3. Characterization

All the synthesized products were analyzed by FT-IR with a PerkinElmer Spectrum 2000 instrument (Perkin-Elmer, San Francisco, CA, USA) provided with an attenuated total reflection (ATR) system. FT-IR transmission measurements were recorded at the range of 4000 to 500 cm^−1^ at a resolution of 4 cm^−1^ averaged over 64 scans. ^1^H-NMR spectra were recorded on a Varian Mercury Plus 400 MHz (Agilent, Santa Clara, CA, USA) using DMSO-d_6_ as a solvent. The exact amount of MWCNTs was determined for all mixtures by thermogravimetric analysis (TGA). TGA was carried out in a nitrogenous environment with TA-Instruments Q50-1182 (Mettler Toledo, Columbus, OH, USA) from 20 to 700 °C at a heating rate of 10 °C/min. Glass transition temperatures (*T*_g_) were determined for all samples using a TA-Instruments Q1000 DSC under N_2_ atmosphere. *T*_g_ was calculated as the point of inflexion in the DSC curve baseline. Two cycles were performed, and heating and cooling rates were set to 10 °C/min throughout the DSC measurements in the range of temperature of −70 °C to 120 °C. The nanocomposite was characterized by high-resolution TEM imaging by an Inspect 50 FEI instrument (FEI, Hillsboro, OR, USA). Samples were analyzed in contrast mode by cryogenic fracture in order to create a surface with exfoliated MWCNTs. Each sample resistance was measured with a Gossen Metrawatt Metrahit 18S multimeter (GMC-Instruments Nederland B.V., NL-3449 JD Woerden, The Netherlands). The ultraviolet-visible-infrared (UV-VIS-NIR) reflectance spectrum was measured by a Cary5000 spectrometer (Agilent) equipped with the Cary Diffuse Reflectance Accessory (DRA). A commercially available 100W IR lamp (Kerbl, Buchbach, Germany) was used for the heating tests. The specimen temperature was measured for the duration of the test by a FLIR™ B50 infrared thermo-camera (FLIR, Wilsonville, OR, USA).

## 3. Results and Discussion

### 3.1. Chemical Modification of Polyketone

All reactions between PKs and the different amino-compounds (Scheme 1) were successfully carried out in the bulk according to the different molar ratios as reported in Table 2. The reaction between PK and PMA was also carried out also under microwave irradiation to increase the reaction rate. In this work, only the results gathered from the use of the microwave apparatus are reported. The formation of the desired modified PKs was confirmed by ^1^H-NMR, FT-IR (Figures S1–S4 in Supplementary Materials), and elemental analysis, and occurred with high conversion efficiency for all different amines that were used. This result validates the robustness and versatility of the Paal–Knorr reaction in providing PKs that are functionalized by primary amines with a different chemical nature.

TMA was used in excess with respect to the other amines to check whether an effect of the concentration of the aromatic pendent groups could be beneficial in the ultimate properties of the nanocomposite.

**Figure 1:**
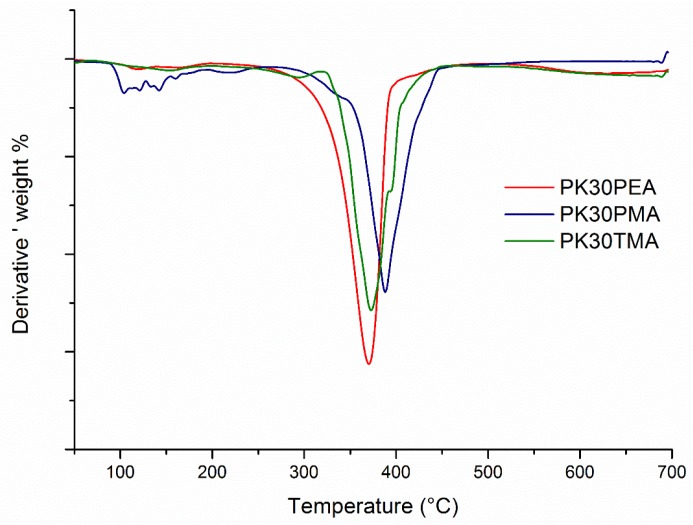
Comparison of the first derivative thermogravimetric analysis (TGA) curves of the different functionalized PKs.

The functionalized PKs were also analyzed by thermogravimetric analysis (TGA) aimed at determining their thermal stability. All polymers remained stable up to ~300 °C (Figure 1), are were therefore suitable for the successive processing for the formation of the nanocomposites. The background noise recorded for the TGA curves just above 100 °C was attributed to the small weight loss (1–2%) caused by moisture evaporation.

It is worth noting that PK30PMA, i.e., the PK functionalized bearing pyrene pendent groups, displayed the highest thermal stability, possibly due to the radical scavenging features of the pyrene ring [41].

### 3.2. Mixing of TMA/PEA/PMA Modified PK30 with MWCNTs

PK nanocomposites were designed to target a flexible, light, and electrically conductive system. In this attempt, all PK30 functionalized polymers were used for the preparation of the MWCNTs nanocomposites, excluding PK30nBuA in view of its relatively low *T*_g_ of −6.4 °C, which renders it unsuitable for the formation of self-standing films. The functionalized PK/MWCNT nanocomposites were prepared by mixing the polymer with different amounts of MWCNTs (Table 1) in toluene. The TGA analysis yields the amount of MWCNTs dispersed in the polymer matrix from the difference in weight between the residuals at 600 °C and confirmed the content introduced in the feed, thus indicating the effectiveness of the blending process.

**Figure 2:**
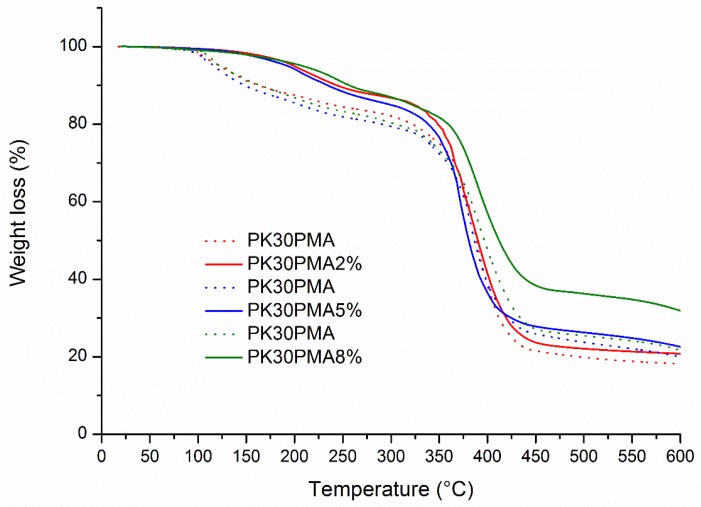
TGA curves of PK30PMA nanocomposites containing the 2, 5, and 8 wt % of MWCNTs.

The TGA curves in Figure 2 showed the typical degradation of functionalized PKs with the loss of the pending moieties at low temperatures (at *T* > 200 °C) followed by the decomposition of the aminated nitrogen heterocycle at about 300 °C, and eventually the severe degradation of the polymeric backbone beyond 350 °C [42].

Another important result gathered from the TGA investigation was the thermal stabilization of the nanocomposites based on PK30PMA. It was worth noting that the combination of the PK30PMA matrix and the scavenging characteristics of MWCNTs postponed the thermal degradation of the nanocomposite by approximately 80 °C. This enhancement in polymer stability was not observed in the other PK30-based samples (Figure S5). The extra stability could be provided to the system by the possible existence of strong and effective interactions between the pyrene moieties and the dispersed MWCNTs, as analogously reported in literature for highly homogeneous polymer nanocomposites based on graphitic fillers [34,35,43].

**Figure 3:**
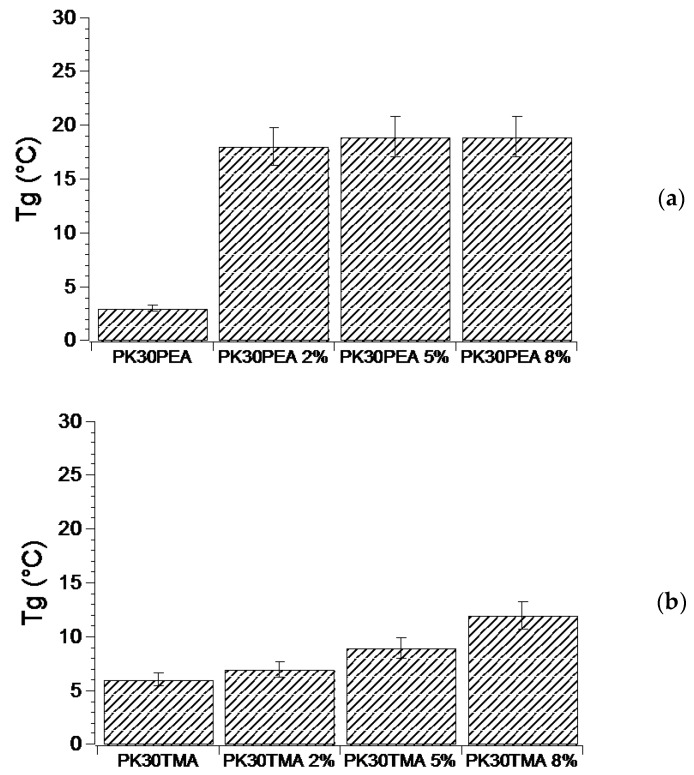

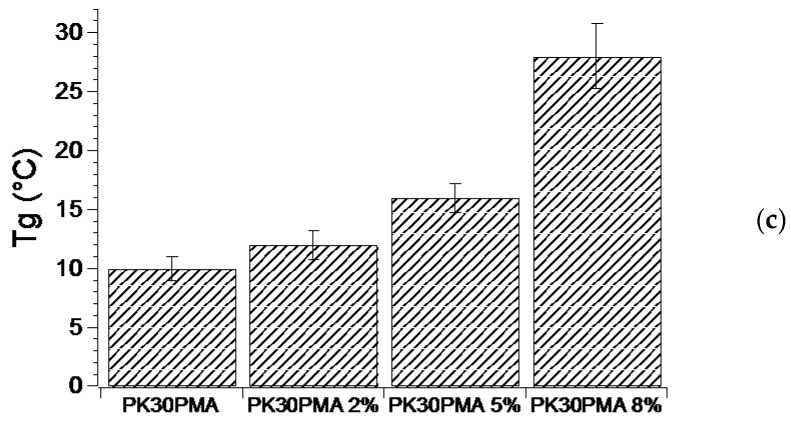
*T*_g_ of the functionalized PK30 polymers ((**a**) PK30PEA, (**b**) PK30TMA, and (**c**) PK30PMA) and of the derived nanocomposites containing different wt % of MWCNTs. The *T*_g_ values were measured by DSC in three temperature cycles from −20 to 180 °C.

Another feature conferred to the PK30 matrix by the presence of the pyrene pendent moieties was the enhanced polymer rigidity, as demonstrated by the increase in the *T*_g_ values of the pristine functionalized polymers by up to 10 °C (Figure 3). The polymer rigidity was also augmented by the addition of MWCNTs, and this behavior was particularly evident for the nanocomposites based on PK30PEA and PK30PMA (Figure 3). Nevertheless, in the case of PK30TMA and PK30PMA, a progressive increase in the *T*_g_ value was found with MWCNTs loading, thus suggesting that the polymer matrix preserves its effective interactions with the graphitic filler even at high concentrations. However, notwithstanding the higher concentration of TMA groups grafted to the PK30 backbone, PK30TMA seemed less efficient than PK30PMA. According to the literature, the increase in *T*_g_ with the MWCNTs content is attributed to the increment in the polymer viscosity [44] due to the interfacial interaction between the polymer chains and the graphitic filler at the molecular level, and is triggered by the presence of functional aromatic groups included in the backbone or as pendent moieties in the polymer chains. This behavior hampers the mobility of the polymer chains and hence increases the *T*_g_ [39,45,46]. As a result, the more powerful the polymer/filler interactions are, the higher the *T*_g_ of the nanocomposite becomes.

**Figure 4:**
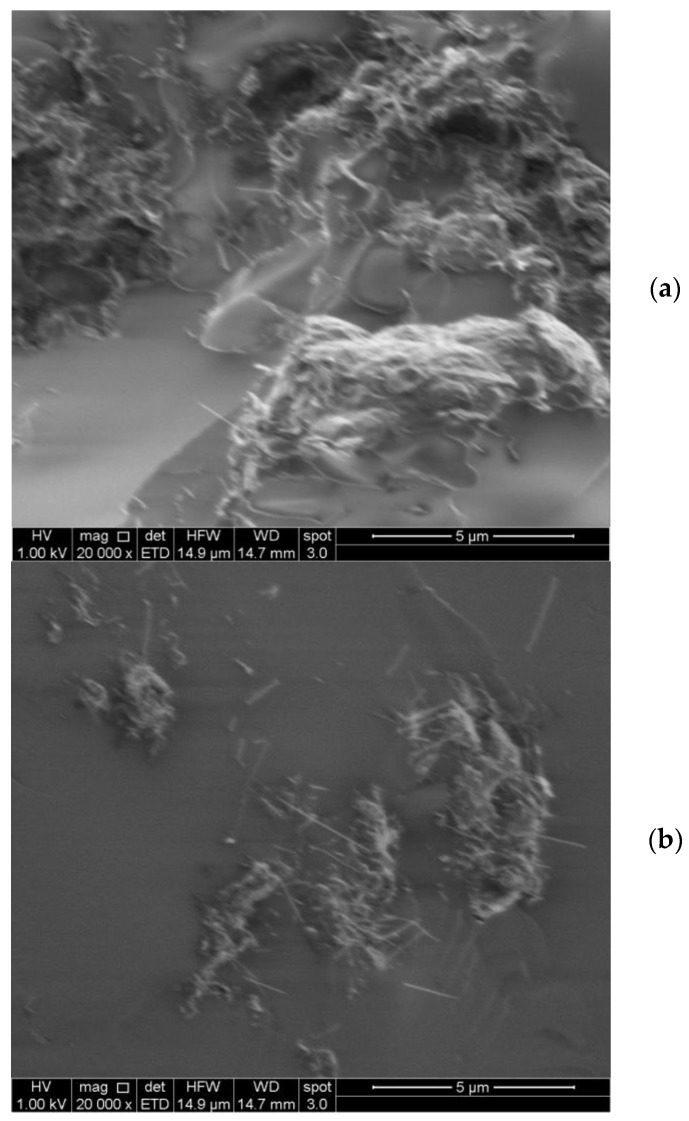

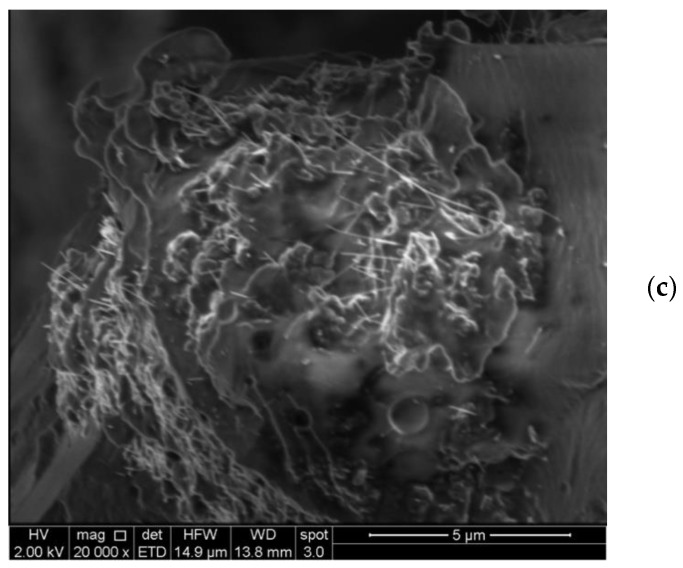
SEM morphological study of PK30PEA (**a**), PK30TMA (**b**), and PK30PMA (**c**) nanocomposites containing 8 wt % MWCNTs.

The PK30PMA, PK30TMA, and PK30PEA samples containing 8 wt % of MWCNTs were then analyzed by SEM (Figure 4).

The micrographs neatly correspond with the results obtained by the DSC investigations. In the PK30TMA and PK30PMA nanocomposites, the MWCNTs appeared more evenly distributed (Figure 4b,c, respectively) without clustering in isolated islands as it had occurred in the PK30PEA matrix (Figure 4a). Moreover, in the case of the PK30PMA nanocomposite, the SEM image clearly displayed the single (unbundled) nanotubes as the dominant species, thus confirming the good interactions between the MWCNTs and the pyrene-functionalized PK matrix. 

### 3.3. Electrical Conductivity Analysis of PK-Based Nanocomposites

**Figure 5:**
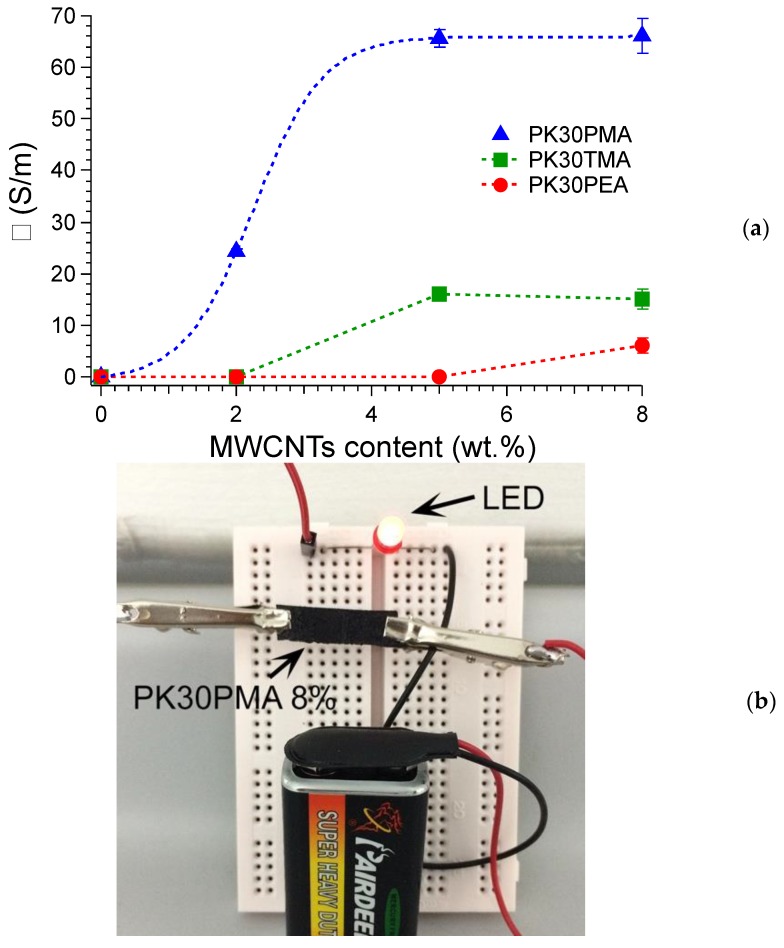
(**a**) Electrical conductivity of the PK30TMA, PK30PEA, and PK30PMA nanocomposites as a function of the MWCNTs loading (wt %); (**b**) photograph of the PK30PMA nanocomposite containing the 8 wt % of MWCNTs connected to an electrical circuit with a red-emitting LED.

The percolation threshold was determined for all three kinds of modified polyketone nanocomposites, in order to identify the optimal percentage of MWCNTs loading required to achieve an interconnecting conductive network throughout the matrix. Annealed nanocomposites at 75 °C for 10 min were studied to observe a better electrical conductivity after melt processing the composite pendent in the literature [24,47]. 75 °C was selected as the annealing temperature since all of the nanocomposite sample became soft, thus potentially favoring MWCNTs reorganization into a more percolative network. The modified polyketone with pyrene shows the lowest percolation threshold (≈2 wt % of MWCNTs) with respect to thiophene and phenyl-modified PK (Figure 5a), possibly thanks to the effective π*–*π interactions with the MWCNTs that were provided by the extended aromatic pendent moieties [48,49]. This result was substantial considering the lower amount of the functional aromatic pendent groups in the PM30PMA with respect to that in PK30TMA. It was worth noting that PK30PMA nanocomposites containing the 5 wt % of MWCNTs reached the considerable electrical conductivity of 65 S/m. Notably, the PK30PMA nanocomposite connected to an electrical circuit with a 9V battery was able to turn on a red-emitting LED (Figure 5b). The low conductivity reached by the PK30PEA nanocomposite even at the highest MWCNTs content confirmed the lesser ability of the phenyl pendent groups in activating a powerful interaction with the filler, which caused the formation of a loose MWCNTs network within the matrix.

**Figure 6:**
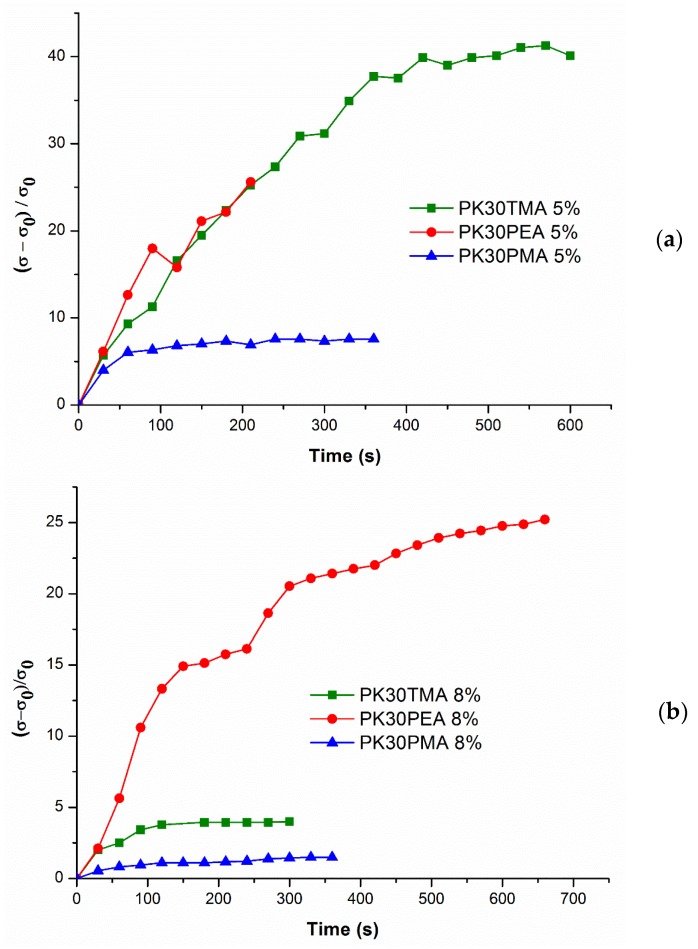
Comparison of the relative conductivity variation of PK30TMA, PK30PEA, and PK30PMA nanocomposites containing the (**a**) 5 wt % and (**b**) 8 wt % of MWCNTs as a function of the annealing time at 75 °C.

Measurements of electrical conductivity were also carried out on fresh samples aimed at determining whether the pristine nanocomposites could be already conductive and to monitor its variation under the annealing conditions (Figure 6). Only the PK30PEA nanocomposite with 5 wt % of MWCNTs was found to be nonconductive in fresh samples. As a consequence, an apparent positive variation of the electrical conductivity was found during the annealing for PK30PEA and for PK30TMA nanocomposites too, i.e., those systems characterized by a higher percolation threshold and a lower electrical conductivity (see Figure 5a). Conversely, the PK30PMA nanocomposites were found to be already well conductive in pristine samples and showed the smallest variations in conductivity with the annealing time. The increase in electrical conductivity was practically negligible for the sample containing the 8 wt % of MWCNTs. At this content, the PK30TMA nanocomposite also showed limited variations in the electric response as a confirmation that the more homogeneous MWCNTs phase dispersion allowed the formation of a more effective and stable percolation network already after nanocomposite processing.

**Figure 7:**
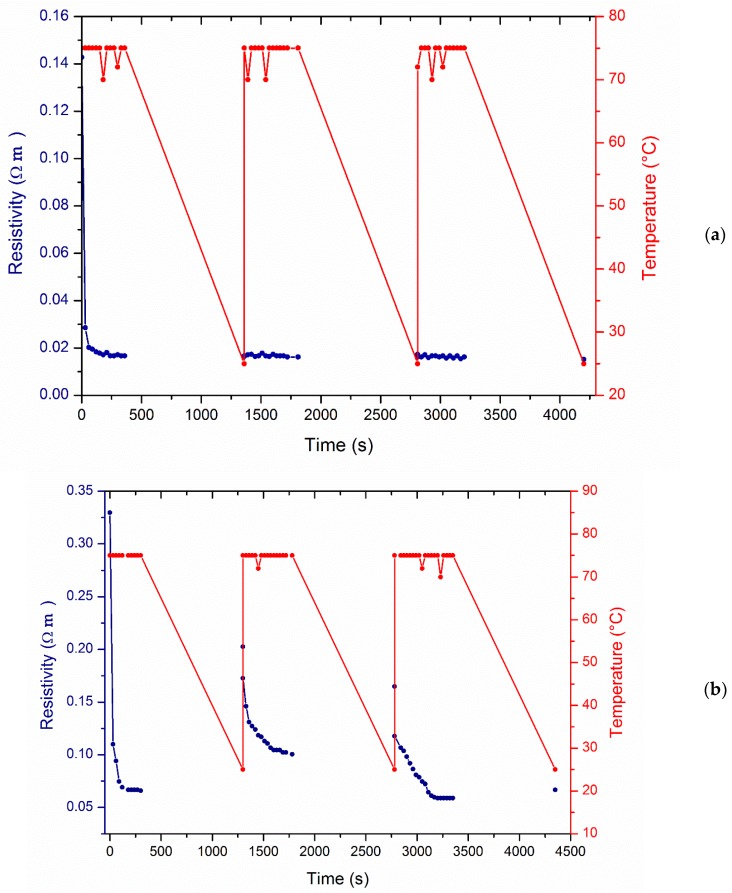
Resistivity changes of (**a**) PK30PMA and (**b**) PK30TMA nanocomposites containing the 8 wt % of MWCNTs as a function of heating–cooling cycles from 25 to 75 °C.

On this basis, the PK30PMA nanocomposite proved to be the system with the highest stability in the electric response during annealing at the softening point. Therefore, it was selected for further investigations aimed at eventually exploring its electric response towards different heating–cooling cycles from 25 to 75 °C. The resistivity of the PK30PMA nanocomposite was plotted as a function of the heating and cooling time and compared to that measured of PK30TMA system (Figure 7). Notably, the resistivity was selected here as it was a more effective parameter than its reciprocal, the electrical conductivity, in providing a more reliable analysis.

The as-prepared PK30PMA nanocomposite demonstrated to be already conductive with a resistivity of about 0.14 Ω∙m. This feature confirms the results gathered from SEM and DSC investigations that evidenced the ability of the PK30PMA in activating effective interactions with the graphitic filler, which in turn allowed their homogeneous and percolative distribution within the polymer matrix. During the first cycle of annealing, the resistivity progressively decreased according to the electric conductivity variation as reported in Figure 6b, and levelled off to a constant value of about 0.02 Ω∙m. No further variations in resistivity were recorded after cooling and, notably, after successive heating–cooling cycles. Conversely, the nanocomposite based on PK30TMA and containing the same 8 wt % of MWCNTs (Figure 7b) but a higher content of aromatic pendent groups also showed a temperature-dependent resistivity for the second and the third heating–cooling cycle, thus suggesting the presence of a percolative network strongly affected by external solicitations such as thermal stress. Moreover, the poor reversibility of the resistivity values between each cycle possibly indicates the flawed phase dispersion of MWCNTs, thus confirming the results from microscopies.

**Figure 8:**
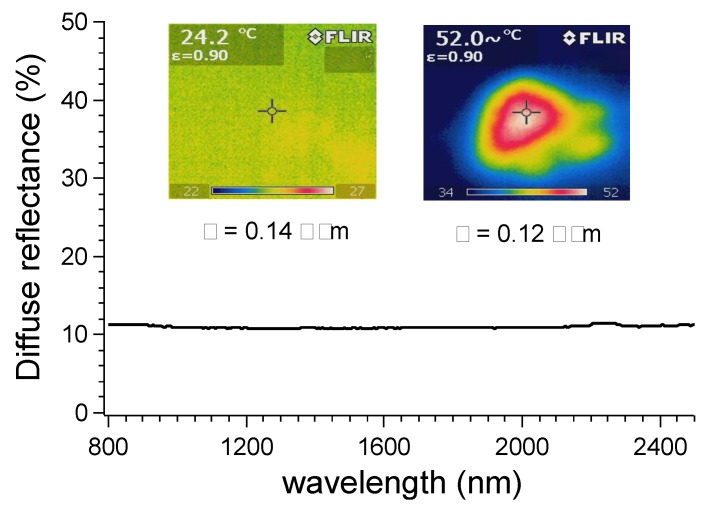
Diffuse reflectance of PK30PMA 8 wt % nanocomposite and thermal images before and after exposure to IR light for 30 min.

Since the nanocomposite was able to absorb heat energy once exposed to IR light due to the presence of MWCNTs [50], the resistivity of the PK30PMA 8 wt % nanocomposite was also monitored during the IR illumination. After 30 min of exposure, the temperature of the sample raised by approximately 30 °C due to the absorption of more than 85% of the NIR radiation (Figure 8).

Notwithstanding the heating up of the sample, a negligible variation of the nanocomposite resistivity was detected, i.e., from 0.14 Ω∙m at 24 °C to 0.12 Ω∙m at 52 °C.

Overall, all the experiments collected evidenced the capability of the pyrene-functionalized PK in supporting the most effective interactions with MWCNTs, their homogeneous phase dispersion, and a stable percolative network useful in providing a constant electric response even to successive cycles of thermal stress.

## 4. Conclusions

Novel thermoplastic PKs were prepared via the Paal–Knorr modification of an alternating aliphatic PK and labelled by aromatic pendent groups to promote the non-covalent functionalization of MWCNTs through effective π–π interactions. The chemical reaction converted the waxy PK into a flexible polymer with tunable *T*_g_ values depending on the nature of the aromatic functionalities. Notably, the highest *T*_g_ and degradation temperature was detected for the pyrene-containing PK30PMA due to the enhanced polymer rigidity and scavenging activity endowed by the extended aromatic nuclei.

Among the investigated systems, PK30PMA was also found to be the most effective polymer in promoting strong interactions with MWCNTs, as revealed by the progressive enhancement of the polymer rigidity with filler loading and the well-exfoliated and undamaged nanotubes network. This feature allowed the pyrene-modified PK to display the lowest percolation threshold of approximately 2 wt % of MWCNTs and a maximum electrical conductivity of 65 S/m that was well above those of the other modified PKs. The PK30PMA nanocomposite was already well-conductive in freshly prepared samples, and its percolative network was proved to be the most stable during thermal and infrared annealing and after different heating–cooling cycles from room temperature to the softening point at 75 °C. Overall, all these data consistently support the use of pyrene as a functionalizing agent for PKs in providing a plastic-conductive network with a moderate concentration of MWCNTs and a very stable electrical response even after external solicitations such as thermal stress. Future approaches will be aimed at better rationalizing the combination of the polymer matrix and its molecular weight, extending the study to conductive fillers of a different nature and aspect ratio.

## Figures and Tables

**Table 1:** Experimental conditions of functionalized PKs mixed with different wt % of multiwalled carbon nanotubes (MWCNTs).

Sample	MWCNTs (%)
PK30TMA	2
PK30TMA	5
PK30TMA	8
PK30PEA	2
PK30PEA	5
PK30PEA	8
PK30PMA	2
PK30PMA	5
PK30PMA	8

**Table 2:** Ratio of amine/PK30 in alimentation, the carbonyl conversion (Χ_CO_), and the amine conversion (Χ_amine_) calculated according to Equations (1) and (2), respectively.

Sample	Ratio Amine/PK30	X_CO_ (%)	X_amine_ (%)
PK30nBuA	0.40	35.00	95.00
PK30PEA	0.40	43.05	98.05
PK30TMA	0.60	58.78	98.78
PK30PMA	0.40	36.82	96.82

## Supplementary Materials

The following are available online at http://www.mdpi.com/2073-4360/10/6/618/s1, Figure S1: (a) ^1^H-NMR and (b) FT-IR spectra of modified pristine PK30 and PK30nBuA, Figure S2: (a) ^1^H-NMR and (b) FT-IR spectra of modified pristine PK30 and PK30TMA, Figure S3: (a) ^1^H-NMR and (b) FT-IR spectra of modified pristine PK30 and PK30PEA, Figure S4: (a) ^1^H-NMR and (b) FT-IR spectra of modified pristine PK30 and PK30PMA, Figure S5: TGA curves of (a) PK30PEA and (b) PK30TMA with 2, 5 and 8 wt. % of MWCNTs.
