# Supplementary material for: Effect of the Polyketone Aromatic Pendent Groups on the Electrical Conductivity of the Derived MWCNTs-Based Nanocomposites
Source: Polymers (Basel). 2018 Jun 5;10(6):618. doi: 10.3390/polym10060618 (PMC6404154; doi:10.3390/polym10060618)
Supplement: Supplementary file 1 [file polymers-10-00618-s001.zip › polymers-313626-SI.pdf]

Supplementary Materials: Effect of the Polyketone Aromatic Pendent Groups on the Electrical Conductivity of the Derived MWCNTs-Based Nanocomposites

Nicola Migliore, Lorenzo Massimo Polgar, Rodrigo Araya-Hermosilla, Francesco Picchioni, Patrizio Raffa and Andrea Pucci

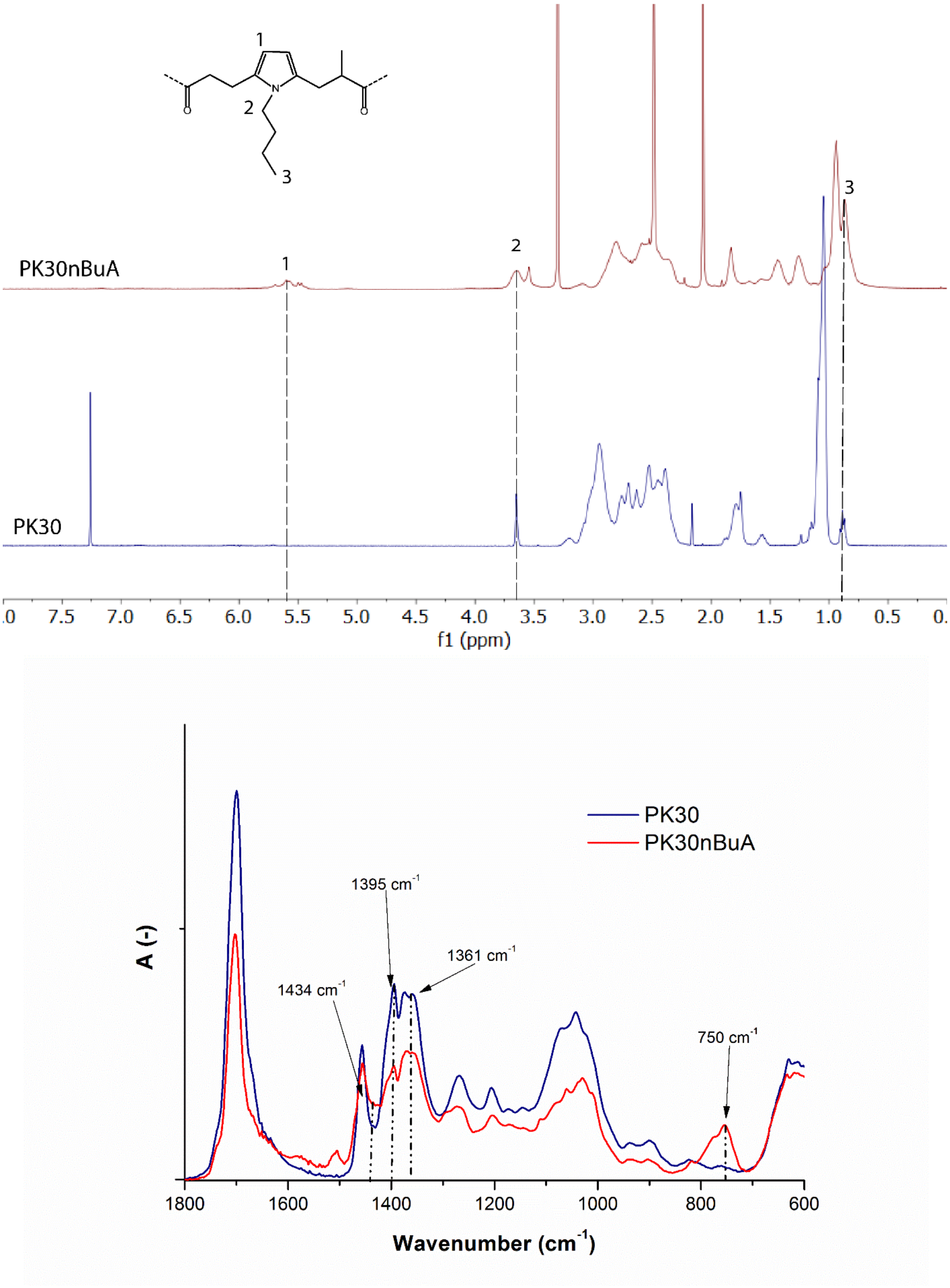

Figure S1: a) ^{1}H-NMR and b) FT-IR spectra of modified pristine PK30 and PK30nBuA. 1395 cm^{-1} (CH_2 twisting) 1361 cm^{-1} (CH_2 wagging), 1434 cm^{-1} (pyrrole ring stretching), 750 cm^{-1} (aromatic C-H out-of-plane aromatic rings)

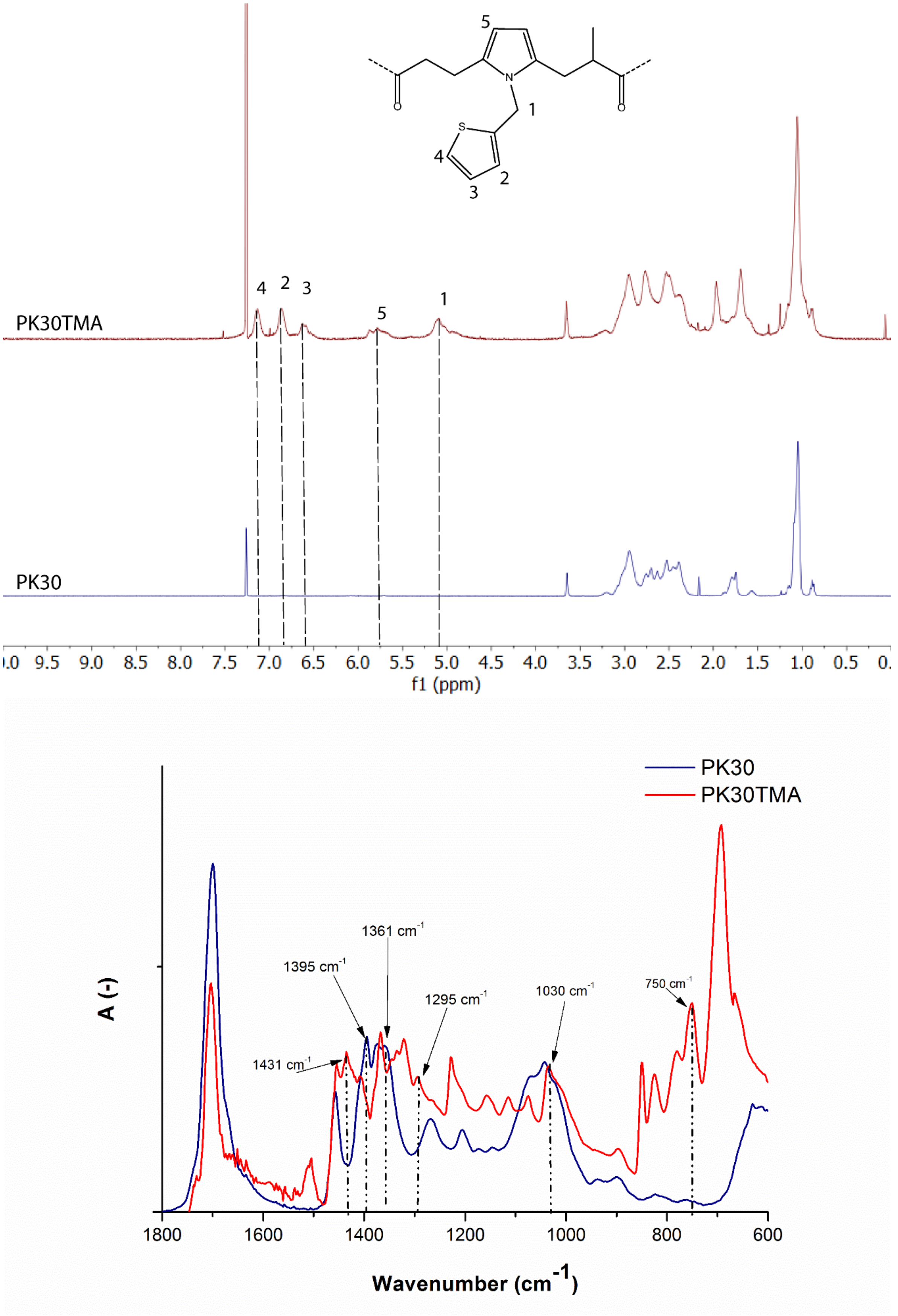

Figure S2: a) ^{1}H-NMR and b) FT-IR spectra of modified pristine PK30 and PK30TMA. 1395 cm^{-1} (CH_2 twisting), 1361 cm^{-1} (CH_2 wagging), 1431 cm^{-1} and 1345 cm^{-1} (pyrrole and thiophene ring stretching), 1073 cm^{-1} (thiophene C-C bending), 750 cm^{-1} (aromatic C-H out-of-plane aromatic rings)

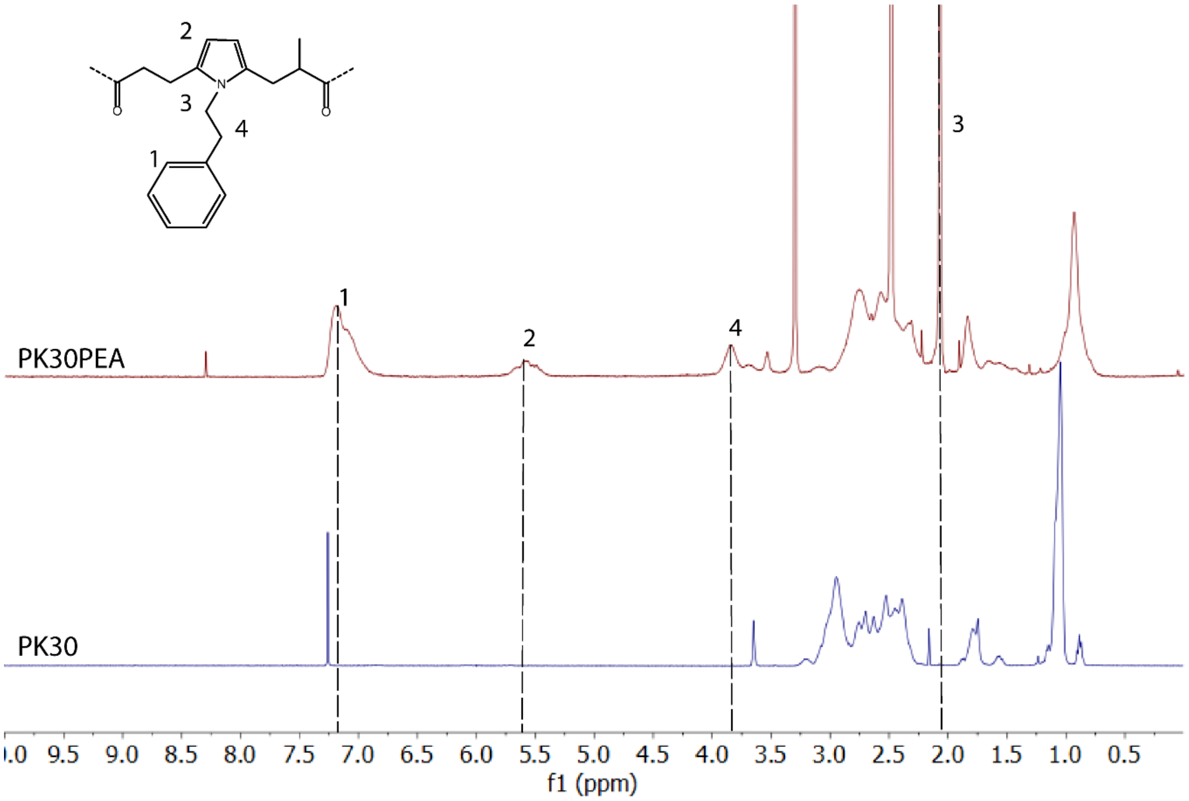

a)

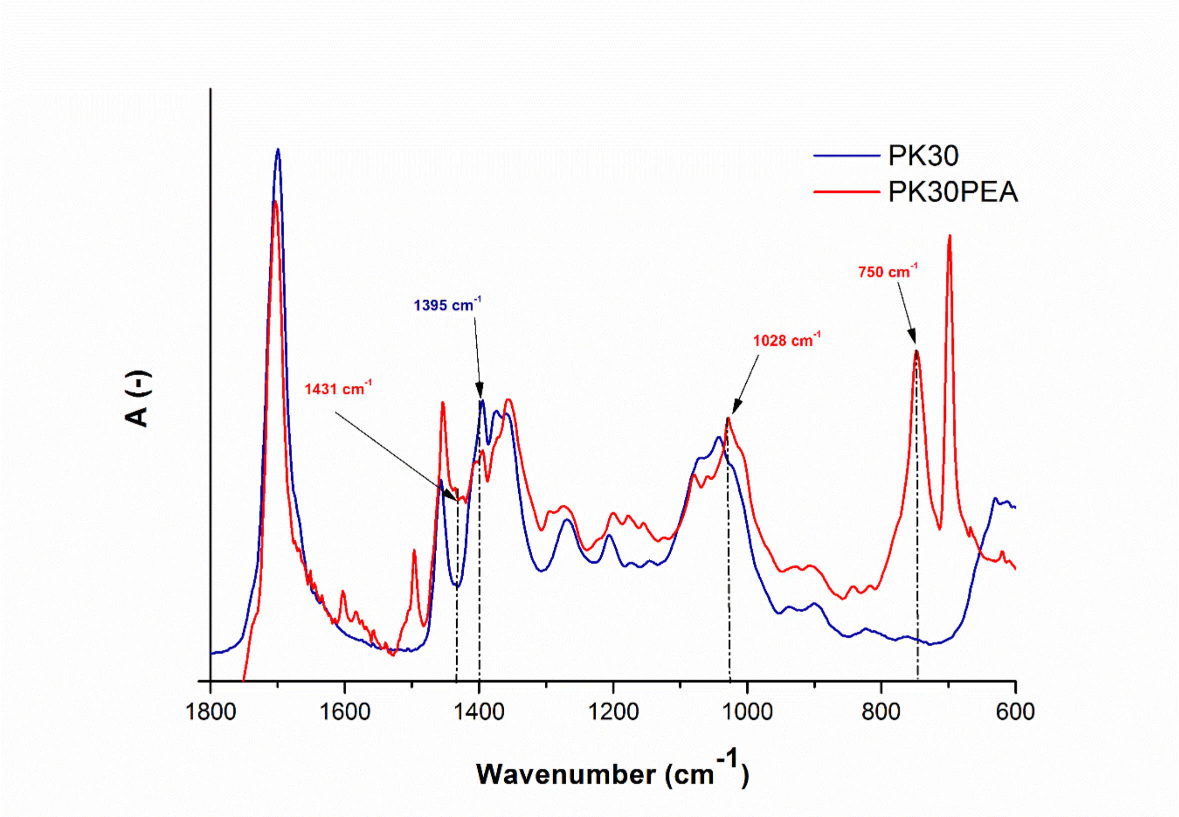

b)

Figure S3: a) ^{1}H-NMR and b) FT-IR spectra of modified pristine PK30 and PK30PEA.
1395 cm^{-1} (CH_2 twisting), 1431 cm^{-1} (pyrrole ring stretching), 1028 cm^{-1} (pyrrole C-C), 750 cm^{-1} (aromatic C-H out-of-plane aromatic rings).

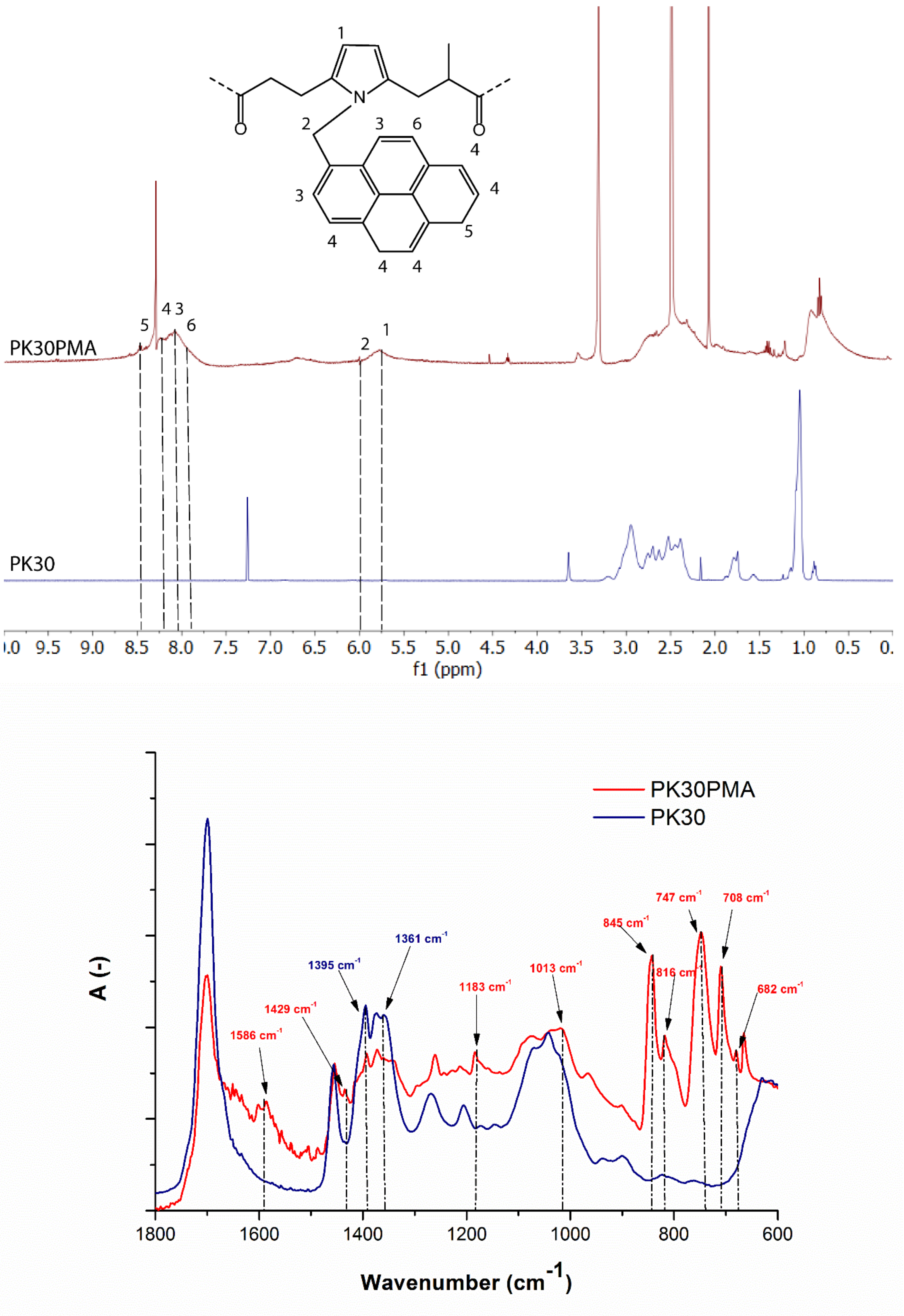

a)

b)

Figure S4: a) ^{1}H-NMR and b) FT-IR spectra of modified pristine PK30 and PK30PMA. 1395 cm^{-1} (CH_2 twisting), 1361 cm^{-1} (CH_2 wagging), 708 cm^{-1} and the moderate absorptions at 1183 and 747 cm^{-1} are assigned to the out-of-plane vibrations of the three adjacent C-H bonds of the pyrene ring and the newly formed C-C bond between pyrene units, respectively. In addition, the strongest peaks at 845 cm^{-1} and the weak peaks at 1429 and 1686 cm^{-1} were assigned to the C=C bonds of the pyrene. Two new peaks at 816 and 682 cm^{-1} are attributed to the two adjacent C–H bonds of the pyrene ring and the newly formed C–C bond between pyrene units, respectively.

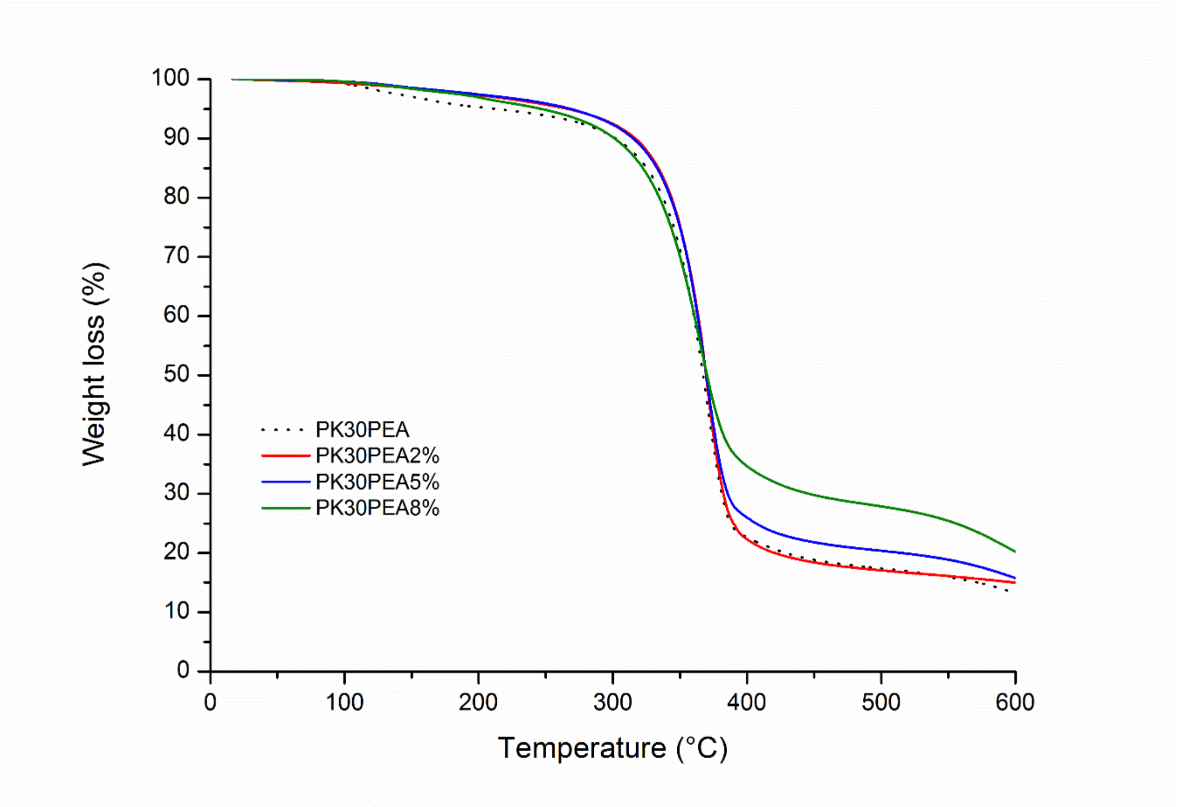

a)

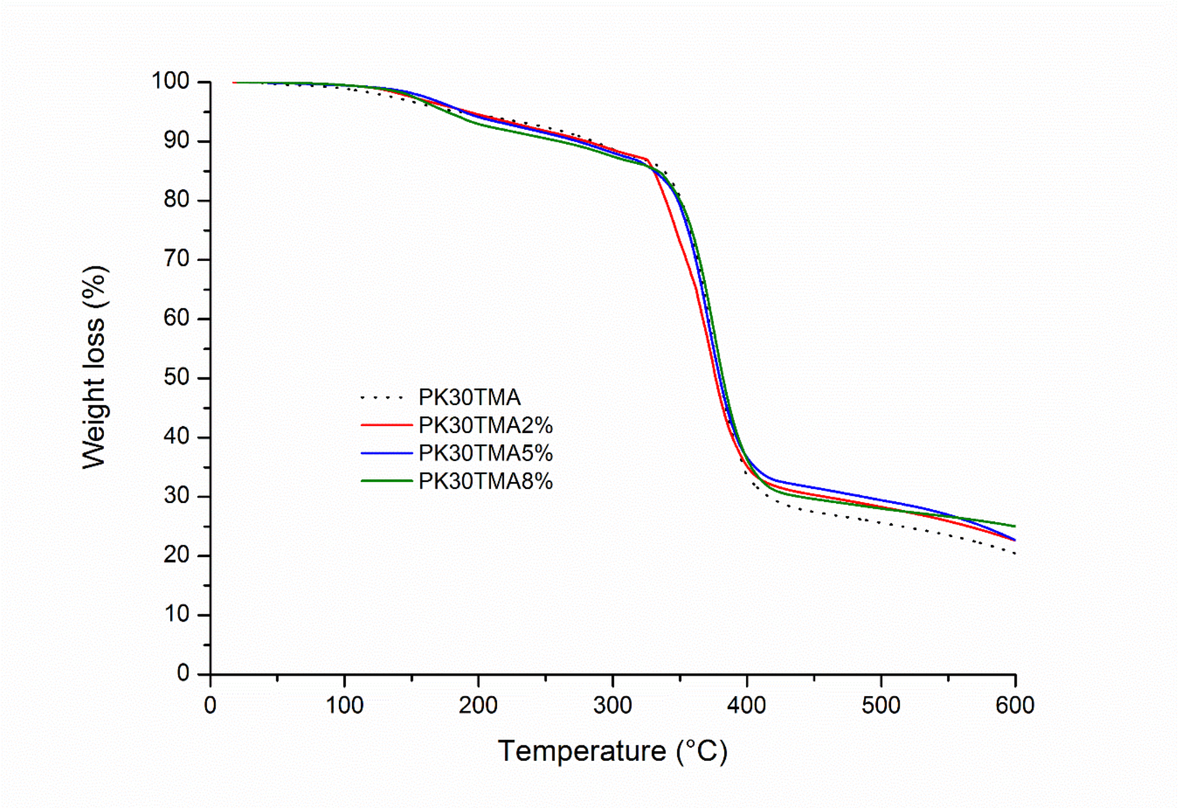

b)

Figure S5: TGA curves of a) PK30PEA and b) PK30TMA with 2, 5 and 8 wt. % of MWCNTs.
